# Insight into diversity change, variability and co-occurrence patterns of phytoplankton assemblage in headwater streams: a study of the Xijiang River basin, South China

**DOI:** 10.3389/fmicb.2024.1417651

**Published:** 2024-08-19

**Authors:** Yuyang Peng, Chuangfeng Wu, Guibin Ma, Haiming Chen, Qinglong L. Wu, Dan He, Erik Jeppesen, Lijuan Ren

**Affiliations:** ^1^Department of Ecology and Institute of Hydrobiology, Jinan University, Guangzhou, China; ^2^Center for Evolution and Conservation Biology, Southern Marine Sciences and Engineering Guangdong Laboratory, Guangzhou, China; ^3^State Key Laboratory of Lake Science and Environment, Nanjing Institute of Geography and Limnology, Chinese Academy of Sciences, Nanjing, China; ^4^Sino-Danish Centre for Education and Research, University of Chinese Academy of Sciences, Beijing, China; ^5^Department of Ecoscience, Aarhus University, Aarhus, Denmark; ^6^Limnology Laboratory, Department of Biological Sciences and Centre for Ecosystem Research and Implementation, Middle East Technical University, Ankara, Türkiye; ^7^Institute for Ecological Research and Pollution Control of Plateau Lakes, School of Ecology and Environmental Science, Yunnan University, Kunming, China

**Keywords:** headwater streams, phytoplankton diversity, region-scale biogeography, assembly processes, variability and vulnerability

## Abstract

Phytoplankton has been used as a paradigm for studies of coexistence of species since the publication of the “paradox of the plankton.” Although there are a wealth of studies about phytoplankton assemblages of lakes, reservoirs and rivers, our knowledge about phytoplankton biodiversity and its underlying mechanisms in mountain headwater stream ecosystems is limited, especially across regional scales with broad environmental gradients. In this study, we collected 144 phytoplankton samples from the Xijiang headwater streams of the Pearl River across low altitude (< 1,000 m) located in Guangxi province, intermediate altitude (1,000 m < altitude <2,000 m) in Guizhou province and high altitude (> 2,000 m) in Yunnan province of China. Our study revealed high phytoplankton diversity in these streams. Freshwater phytoplankton, including cyanobacteria, Bacillariophyta, Chlorophyta, Rhodophyta, Chrysophyta, Euglenophyta, Glaucophyta, Phaeophyta and Cryptophyta, were all detected. However, phytoplankton alpha diversity exhibited a monotonic decreasing relationship with increasing altitude. High altitudes amplified the “isolated island” effect of headwater streams on phytoplankton assemblages, which were characterized by lower homogeneous selection and higher dispersal limitation. Variability and network vulnerability of phytoplankton assemblages increased with increasing altitudes. Our findings demonstrated diversity, variability and co-occurrence patterns of phytoplankton assemblages linked to environmental factors co-varying with altitude across regional scales.

## 1 Introduction

Since the publication of the “paradox of the plankton” (Hutchinson, 1961), phytoplankton has been considered a useful paradigm system for exploring the mechanisms maintaining high biodiversity. Environmental factors such as temperature and nutrient availability have been demonstrated to influence phytoplankton biodiversity (Irigoien et al., 2004; Morán et al., 2010; Vallina et al., 2014; Verbeek et al., 2018; Chang et al., 2022). Although there is wealth of scientific information about freshwater phytoplankton assemblages of lakes, reservoirs and rivers (Caputo et al., 2008; Naselli-Flores, 2014; Lu and Gan, 2015; Hu et al., 2017; Yu et al., 2023; Stanković et al., 2024), our knowledge about phytoplankton biodiversity and its underlying mechanisms in mountain stream ecosystems is limited (Wang et al., 2016, 2021; de Oliveira et al., 2020; Fodelianakis et al., 2022). Biodiversity of mountains have been studied intensively for more than 250 years, originating with Linnaeus (1781) and Von Humboldt (1849). However, uncovering the mechanisms underlying the extraordinary biodiversity in mountain areas remains a great challenge (Rahbek et al., 2019a; Trew and Maclean, 2021). It is unclear whether the general high abundance, small size, fast population growth and long-range dispersal of algae is sufficient to avoid altitude constrains in mountain stream ecosystems.

Mountain stream ecosystems, including headwater streams, are downstream sources for new propagules, which play an important role in maintaining region-scale aquatic phytoplankton biodiversity and ecosystem services (Sharma et al., 2016; de Oliveira et al., 2020; Zeng et al., 2023), but many are under threat due to ongoing environmental changes, such as habitat loss, pollution, mine drainage, and global warming (Trew and Maclean, 2021; Schmeller et al., 2022). Understanding the altitudinal patterns of phytoplankton assemblages and their underlying mechanisms in such ecosystems is, therefore, essential to predict their response to future environmental changes and to develop water management policies to mitigate the pressures of environmental changes on biodiversity loss (Cardinale et al., 2012; Trew and Maclean, 2021; Schmeller et al., 2022). In the Pearl River Basin of China, headwater streams cover a broad altitudinal gradient, spanning from approximately 320 to 2,220 m (Liu and Han, 2021b), thus providing an ideal altitudinal gradient for a study of phytoplankton biodiversity patterns. In the largest branch of the Pearl River Basin, the Xijiang River area, mountains or hills may contribute to local coexistence of diverse phytoplankton in its headwater ecosystems in three different ways: (i) by creating heterogeneous climatic habitats for phytoplankton within narrow geographical ranges; (ii) by functioning as dispersal barriers to neighboring “isolated island” headwater streams; (iii) by being a modifier of environmental, hydrological and mineralogical conditions (Vetaas et al., 2019; Rahbek et al., 2019b). Climatic, environmental, hydrological and mineralogical fluctuations across altitudinal gradients might result in diverse non-equilibrium dynamics of phytoplankton in local headwater streams (Naselli-Flores et al., 2003a,b). The diversity of phytoplankton in headwater streams of the Xijiang River area might thus expectedly be characterized by higher coexistence at lower altitudes, where environmental filtering and dispersal barriers may be weaker than any other altitudinal areas (Stomp et al., 2011; Vetaas et al., 2019). In addition, the heterogeneous climatic, environmental, hydrological and mineralogical conditions along the altitudinal gradients may alter the influence of the four key processes that control the biodiversity of phytoplankton on mountains or hills: speciation, dispersal, selection and drift (Rahbek et al., 2019b; Wang et al., 2021, 2022; Pritsch et al., 2023). For instance, seasonal changes in environmental variables at higher altitudes may lead to the loss of phytoplankton species by selecting phytoplankton taxa with a broad range of environmental tolerance (Currie et al., 2004). Temperature, which has direct linkages with altitude, might also impact phytoplankton biodiversity through impacting their physiological metabolisms (Brown et al., 2004; Allen and Gillooly, 2007; Gillooly and Allen, 2007; Stomp et al., 2011). In addition, as precipitation is lower at higher altitudes in the Xijiang River area (Zeng and Han, 2020a,b), the dispersal rates of phytoplankton by atmospheric water circulation might be limited, resulting in an enhanced “isolated island” effect of mountain headwater streams on phytoplankton assemblages. Phytoplankton assemblages in headwater streams might thus exhibit higher variability at higher altitudes. Diversity loss and increased variability of phytoplankton assemblage at higher altitudes might furthermore cause higher network vulnerability of the co-occurrence of phytoplankton to climatic, environmental, hydrological and mineralogical fluctuations. In a co-occurrence network, vulnerability is a measure of the relative contribution of the phytoplankton taxa to the global efficiency. It is accompanied by various network indices, including node or link quantity, network components and heterogeneity, keystone species and modularity (Dunne et al., 2002; Grilli et al., 2016; Yuan et al., 2021). Network vulnerability is an important index that can provide information on how fast the consequence of environmental/ecological fluctuations traverse to parts or the entire co-occurrence network (Yuan et al., 2021).

**Figure 1:**
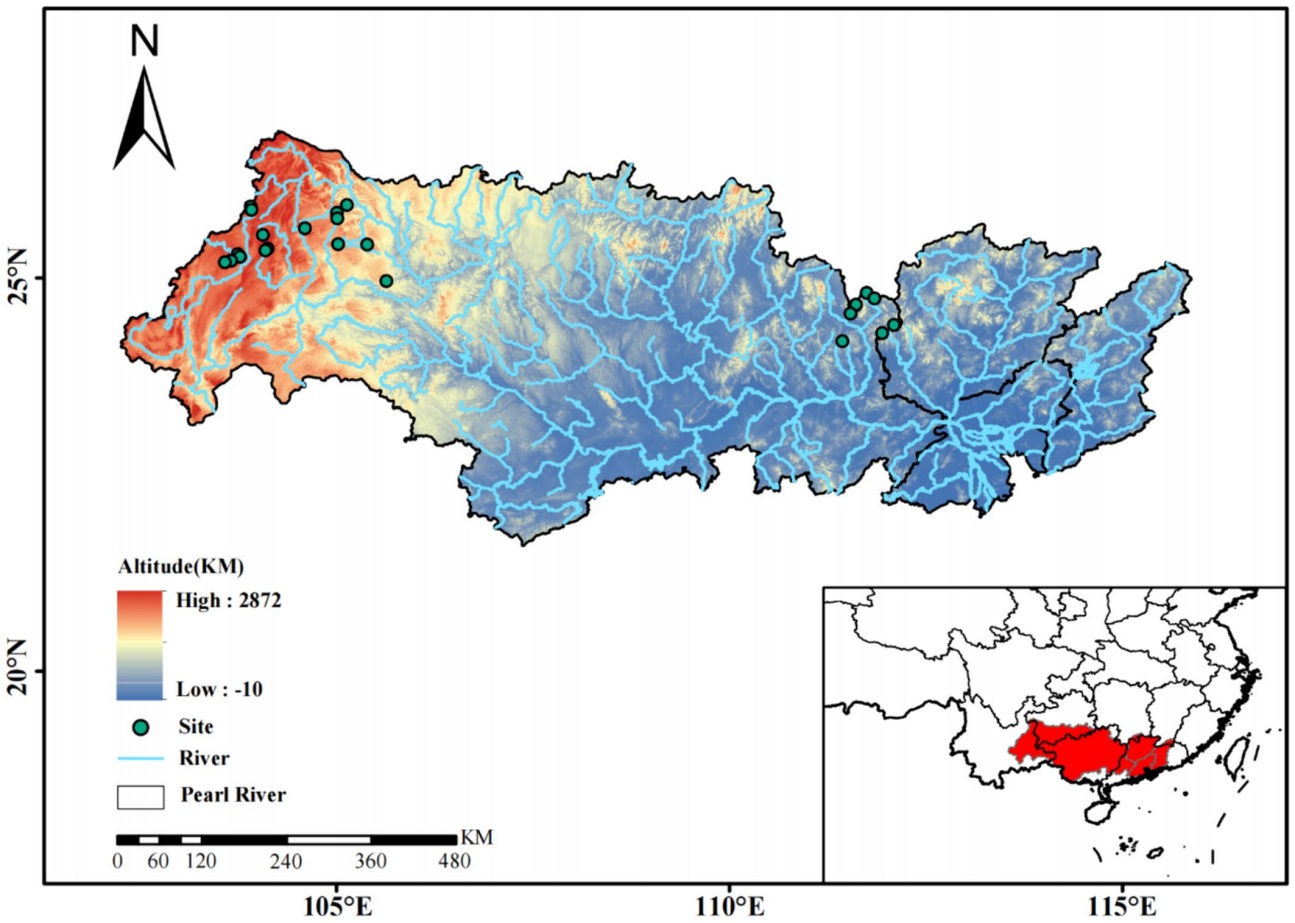
The location of sampling sites in headwater streams in the Xijiang River basin, South China.

To explore species coexistence of phytoplankton, we constructed a community assembly framework (Ning et al., 2020) and co-occurrence networks (Yuan et al., 2021) in headwater streams to elucidate the biodiversity patterns of phytoplankton along an altitudinal gradient (Figure 1). Prior research shows that dispersal limitation, homogenizing dispersal, homogeneous and heterogeneous selection, and drift play different roles in controlling regional-scale biodiversity and co-occurrence patterns of phytoplankton (Guelzow et al., 2017; Klais et al., 2017; Kruk et al., 2021). More substantial seasonal changes, lower temperatures, and higher UV radiation at higher altitudes may impose stronger homogeneous or heterogeneous selection on phytoplankton assemblages. Conversely, lower immigration rates at higher altitudes potentially result in stronger dispersal limitation of phytoplankton among headwater streams. Changed balance of community assembly processes along altitude gradient may have influences on the patterns of diversity, variability and network vulnerability of phytoplankton in headwater streams. Therefore, we hypothesized that in headwater streams in the Xijiang River area: (1) “isolated island” effect of mountain headwater streams on phytoplankton assemblage might be enhanced by altitudes; (2) coexistences of diverse phytoplankton would occur at low altitudes, with lower variability and lower vulnerability of the co-occurrence phytoplankton taxa at lower altitudes; (3) the phytoplankton assemblage along the altitude gradient would be shaped by the balance of selection, dispersal and stochastic drift. To test these three hypotheses, we used high-throughput sequencing to study 144 phytoplankton samples collected in the Xijiang headwater streams of the Pearl River in southern China. The locations covered a broad gradient of altitude, spanning from approximately 320 to 2,220 m (Zeng and Han, 2020a; Liu and Han, 2021a), which provides an ideal altitudinal gradient for the study of phytoplankton biodiversity patterns.

## 2 Materials and methods

### 2.1 Study area at the Xijiang River

The Zhujiang River (Pearl River), located in South-East China, is the second largest river by discharge or the third largest river by length (approximately 2,300 km) in China (Zeng and Han, 2020a,b). Among the four parts of the Pearl River (the Xijiang River, the Beijiang River, the Dongjiang River, and the Zhujiang delta water system), the Xijiang River is the largest tributary of the Pearl River and is famous for its karst landform (Liu and Han, 2021b). The Xijiang River originates in the Yunnan-Guizhou plateau (Maxiong mountain) and then flows through Yunnan, Guizhou, Guangxi and Guangdong Provinces and discharges into the South China Sea (Liu and Han, 2021a,b). There are mountains and hills with altitudes ranging from approximately 300 to 2,300 m in the Xijiang River area. The soil in the region, consists Precambrian metamorphic rocks and Quaternary fluvial sediments (Liu and Han, 2021a; Liu et al., 2022). Permian and Triassic carbonate bedrocks dominate in the upper headwater of the Xijiang River basin, and these Karst geomorphologic phenomena are well developed (Li et al., 2020). The lower headwater of the river basin is primarily characterized by siliceous sedimentary rocks and magmatic and metamorphic rocks (Liu and Han, 2021b). The river is affected by the sub-tropic monsoon climate, with an annual mean temperature ranging from 14°C to 22°C and the annual mean rainfall between 1,200 and 2,200 mm/year (Zeng and Han, 2020b; Liu and Han, 2021b). Precipitation is concentrated in the wet season (April to September temporally) and decreases spatially toward the west (Zeng and Han, 2020a,b).

### 2.2 Sample collection and analysis

From November 2020 to January 2021, 144 phytoplankton samples were collected at 24 stations along the headwater streams (54 samples in Yunnan province, 42 samples in Guizhou province, 48 samples in Guangxi province) (Figure 1). These headwater streams are the source streams at the initiation points of all Xijiang River networks. These headwater streams have small size with an average depth of 0.28 m and an average flow velocity of 0.045 m·s^−1^. At each station, phytoplankton samples (1 to 2 L water, 3 replicates) were collected from surface waters. We separated the samples into cell size >3 μm (primarily micro- and nanophytoplankton) and sizes ranging from 0.2 to 3 μm (primarily picophytoplankton) by filtering the samples through 3-μm-pore-size Isopore filters (Millipore, Billerica, MA, United States), followed by filtration through 0.2-μm-pore-size Isopore filters (Millipore, Billerica, MA, United States). The filters were stored at −80°C for further analyses.

Dissolved oxygen (DO), pH, conductivity and oxidation–reduction potential (ORP) were measured *in situ* using a YSI multiparameter probe (YSI Proplus, Yellow Springs, OH, United States). Depth of the stream (depth) and flow velocity (velocity) were also measured *in situ*. Monthly mean temperature (MMT) was obtained by extracting layer information using ArcGIS. Ammonium (NH_4_^+^), nitrate (NO_3_^−^), nitrite (NO_2_^−^), total nitrogen (TN), total phosphorus (TP), phosphate (PO_4_^3−^), silicate (SiO_3_^2−^), total content of Fe^3+^ (Fe^3+^), total content of Mn^2+^ (Mn^2+^), total content of Ca^2+^ (Ca^2+^), total content of Mg^2+^ (Mg^2+^), total organic carbon (TOC) and chlorophyll *a* (Chla) were measured in the laboratory according to standard methods (Greenberg et al., 1992).

### 2.3 DNA extraction, amplification, sequencing and data processing

Genomic DNA was extracted using the DNeasy PowerWater DNA Isolation Kit (QIAGEN, Hilden, Germany). The V4 hyper-variable region of the 16S rRNA genes was amplified with primers 515F- (5′- GTGCCAGCMGCCGCGGTAA−3′) and 806R (3′- GGACTACHVGGGTWTCTAAT−5′). To distinguish the samples in one Illumina sequencing run, specific 12-mer tag was added to the 5′ end of each primer of each DNA sample. Three replicates of each sample were PCR amplified in a 50 μL reaction mixture containing 25 μL 2x PCR Premix Taq, 10 mM of each primer, 60 ng of genomic DNA and 20 μL of nuclease-free water. The cycling conditions of PCR included 94°C for 5 min, followed by 30 cycles of denaturation at 94°C for 30 s, annealing at 52°C for 30 s, extension at 72°C for 30 s and a final extension at 72°C for 10 min. Positive amplicons were quantified using the PicoGreen dsDNA assay kit (Invitrogen Corporation, Carlsbad, CA, United States) and purified with Zymo’s Genomic DNA Clean and Concentrator kit (Zymo Research Corporation, Irvine, CA, United States). Finally, the amplicons were sequenced using the Illumina HiSeq PE250 platform at Novogene Bioinformatics Technology Co., Ltd. (Beijing, China).

Raw reads of the 16S rRNA gene sequences were analyzed using QIIME 2 (Bolyen et al., 2019). Briefly, (1) demultiplexed samples were imported and exported as a single QIIME 2 artefact file; (2) the sequences were trimmed with cutadapt implemented in QIIME2; (3) dada2 was used to filter, de-replicate, detect reference-free chimera, merge paired-end reads and to cluster the combined sequences to ASV (Amplicon Sequence Variant) table (Callahan et al., 2017); (4) all 16S ASVs identified as chloroplast were picked out from the overall ASV table; (5) chloroplast 16S ASVs were then taxonomically assigned at 90% nucleotide identity against the QIIME 2 classify-sklearn plugin (Decelle et al., 2015; Duerschlag et al., 2022); (6) to reduce errors in the sequencing process, ASVs present in less than three samples were discarded in the subsequent analyses (Needham et al., 2018).

### 2.4 Null model analysis and co-occurrence network construction

To assess the community assembly, the framework of community assembly mechanisms by phylogenetic-bin-based null model analysis (iCAMP) was performed over the whole phytoplankton sample set, including 144 samples, using iCAMP in the R statistical computing environment (Ning et al., 2020).^1^ We then got the relative importance of heterogeneous selection, homogeneous selection, dispersal limitation, homogenizing dispersal and drift, and other fractions for each sample.

A co-occurrence network was constructed where we filtered out the low abundance (relative abundance <0.005) and low frequency (presence <3 samples) taxa before calculating correlation coefficients between any pairwise ASVs. Further, the network adjacency matrix was obtained with a Spearman’s correlation coefficient (r) higher than 0.7 and significance (*p*) < 0.05. The network adjacency matrix was visualized in Gephi (version 0.9.2). The nodes of each network were classified into different topological roles according to within-module connectivity (Zi) and among-module connectivity (Pi) values, including module hubs (highly connected nodes within modules, Zi ≥ 2.5, Pi <0.62), network hubs (highly connected nodes within entire network, Zi ≥ 2.5, Pi ≥0.62), connectors (nodes that connect modules, Zi < 2.5, Pi ≥0.62) and peripherals (nodes connected in modules with few outside connections, Zi < 2.5, Pi <0.62) (Kougioumoutzis et al., 2014). To assess network stability, we calculated network vulnerability based on the codes proposed by Yuan et al. (2021). The network vulnerability means the relative contribution of the node to the global efficiency. The vulnerability of a network is indicated by the maximal vulnerability of nodes in the network. In ecological networks, it can provide information on how fast the consequence of biological/ecological events traverse to parts or the entire network (Yuan et al., 2021).

### 2.5 Statistical analyses

The diversity indexes including alpha diversity (ASV richness), beta diversity (Bray-Curtis’s dissimilarity within each group) and gamma diversity (ASV richness), were calculated using vegan in R. Pearson correlations and principal component analysis (PCA) were performed to depict the relationships among all investigated environmental variables using corrgram (Friendly, 2002) and vegan (Oksanen et al., 2017) in R, respectively. The phytoplankton structure based on Bray-Curtis’s distance was visualized using non-metric multidimensional scaling (NMDS). To test for significant differences between phytoplankton structure in various altitudinal groups, permutational multivariate analysis of variance using distance matrices (PERMANOVA) was carried out based on Bray-Curtis’s dissimilarity using vegan in R. To analyze the taxa-location relationships, a venn network was constructed in R using the data on phytoplankton taxa and the location groups. Significant differences in phytoplankton beta diversity (i.e., phytoplankton variability) among the different groups were tested by permutation test and Tukey multiple comparisons for homogeneity of multivariate dispersions using vegan in R. A permutation multivariate analysis of variance (permutation MANOVA), followed by a pairwise permutation t test, was performed to analyze the significant differences of community assembly processes in groups of altitudes using the RVAideMemoire package in R (Hervé, 2021). Relationships between phytoplankton composition and investigated environmental variables were assessed by Mantel statistics with 999 permutations and canonical correlation analysis (CCA) with an automatic stepwise model using permutation tests in vegan in R. Multiple regression on distance matrices (MRM) was conducted to investigate the relationships of phytoplankton composition with environmental (E.) and spatial (S.) factors and their mixed effect (Mix.). The sampling maps were generated based on an open-access Google satellite map using ArcGIS (version 10.8).

## 3 Results

### 3.1 Environmental properties in the headwater streams

Principal component analysis (PCA) revealed that the environmental properties could be reduced to two principal components (PC1 and PC2) that explained 25.1 and 18.9% of the variation, respectively (Supplementary Figure S1). Altitude had the highest correlation with PC1, followed by monthly mean temperature (MMT) (Supplementary Figure S1). The altitude of the sampling sites ranged from 320 to 2,220 m, whereas the MMT of the sampling sites varied between 9.8 and 17.2°C. According to the altitude, the 72 sampling sites were categorized into three groups, including low altitude (LA, altitude <1,000 m) located in Guangxi province, intermediate altitude (MA, 1,000 m < altitude <2,000 m) in Guizhou province and high altitude (HA: altitude >2,000 m) in Yunnan province.

Altitude was negatively correlated with MMT, water velocity and the aquatic concentration of PO_4_^3−^ and Chla (Pearson correlation: r > −0.25, *p* < 0.05, Supplementary Figure S2) and positively linked to the concentration of Ca^2+^, Mg^2+^, conductivity, TN and NO_3_^−^ (Pearson correlation: r > 0.34, *p* < 0.05, Supplementary Figure S2). Significant positive correlations were also observed among Ca^2+^, Mg^2+^, Fe^3+^, Mn^2+^, conductivity, NO_2_^−^ and NH_4_^+^ (Pearson correlation: r > 0.60, *p* < 0.05, Supplementary Figures S1, S2). The concentration of Chla was positively linked to the concentrations of TP, PO_4_^3−^ and MMT (Pearson correlation: r > 0.28, *p* < 0.05, Supplementary Figure S2) and marginally correlated with the concentration of Fe^3+^ (Pearson correlation: r = 0.21, *p* < 0.1, Supplementary Figure S2).

### 3.2 The alpha and gamma patterns of phytoplankton assemblages in the Xijiang headwater streams

**Figure 2:**
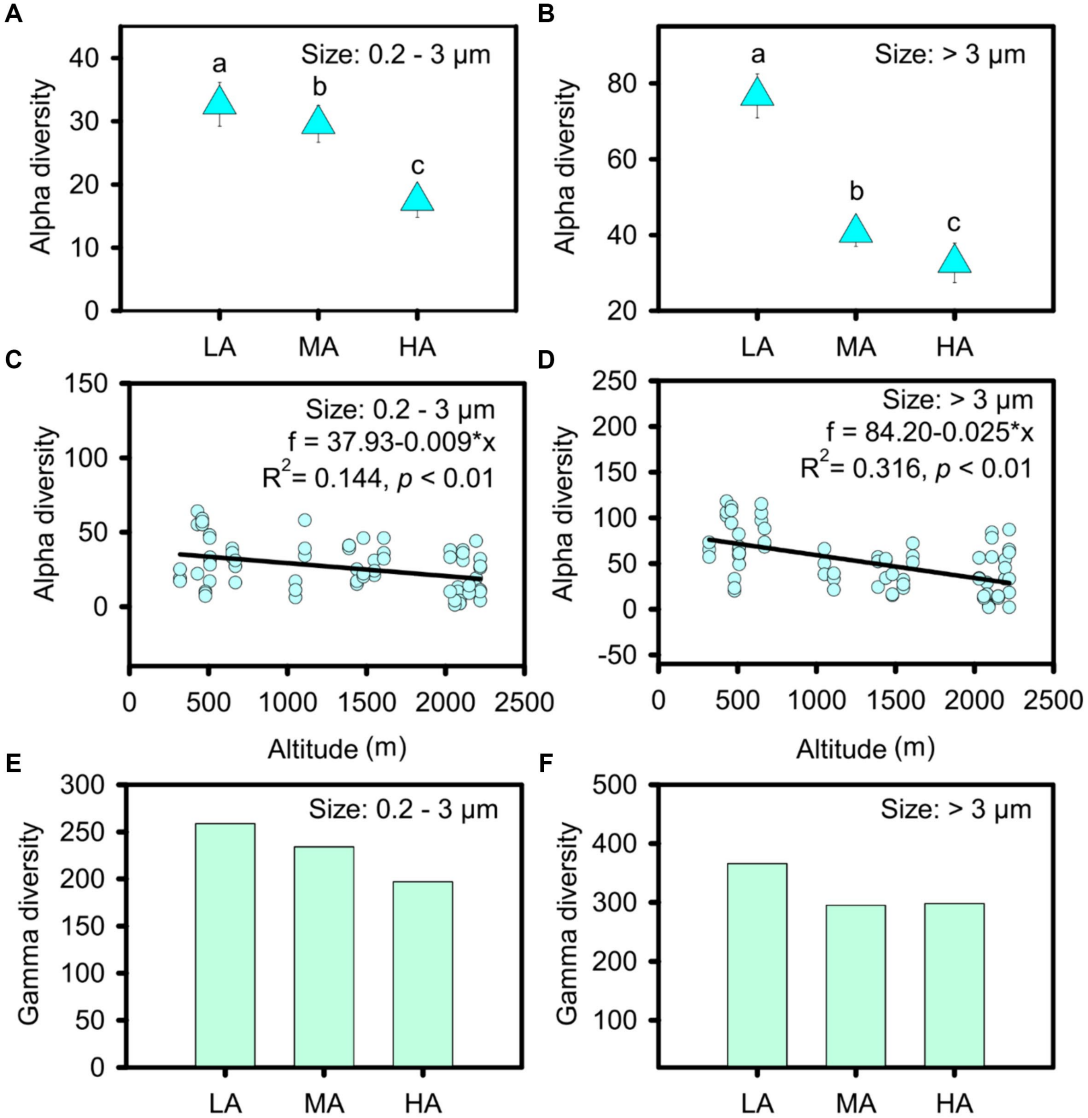
Alpha diversity **(A–D)** and gamma diversity **(E,F)** of the picophytoplankton (size: 0.2–3 μm) and micro- and nanophytoplankton (size >3 μm) communities in the three altitude groups. LA, altitude <1,000 m; MA, 1,000 m < altitude <2,000 m; HA, altitude >2,000 m. Significant differences (*p* < 0.05) among groups are indicated by different alphabetic letters above the bars.

In total, we obtained 413 ASVs in the streams, belonging to nine phyla: cyanobacteria, Bacillariophyta, Chlorophyta, Rhodophyta, Chrysophyta, Euglenophyta, Glaucophyta, Phaeophyta and Cryptophyta (Supplementary Figure S3). However, the nine phyla were found for both cell sizes (Supplementary Figure S3). Micro- and nanophytoplankton were primarily composed of *Microcoleus* and *Bacillariophyceae*, whereas picophytoplankton mainly included *Microcoleus*, *Pseudanabaena* and *Chamaesiphon* (Supplementary Figure S3). Compared with picophytoplankton, a significantly higher alpha diversity was observed for micro- and nanophytoplankton (t test: t = −5.667, *p* < 0.001). When relating alpha diversity to altitude, we found that the alpha diversity of both picophytoplankton and micro- and nanophytoplankton’s decreased gradually with increasing altitudes (picophytoplankton: R^2^ = 0.144, *p* < 0.01; micro- and nanophytoplankton: R^2^ = 0.316, *p* < 0.01) at a rate of 9 ASV per km for picophytoplankton and 25 ASV per km for micro- and nanophytoplankton (Figures 2C,D). In three altitude groups, including low altitude (LA, altitude <1,000 m), intermediate altitude (MA, 1000 m < altitude <2000 m) and high altitude (HA: altitude >2000 m), higher alpha diversity was observed at lower altitudes for both picophytoplankton (ANOVA test: *F* = 7.551, *p* < 0.01) and micro- and nanophytoplankton (ANOVA test: *F* = 21.93, *p* < 0.01) (Figures 2A,B).

**Figure 3:**
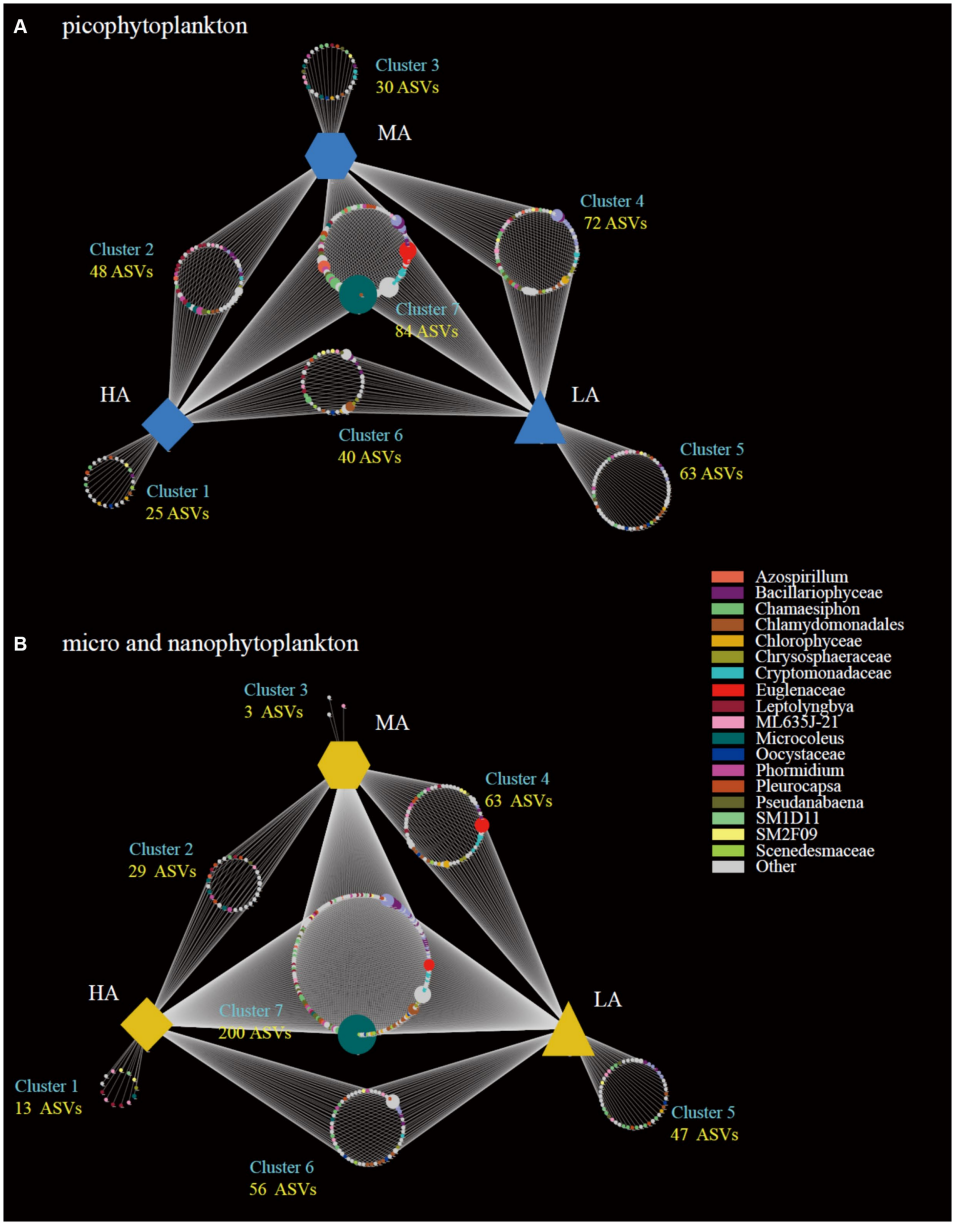
Bipartite association networks showing the associations between the three altitude groups and the significantly associated ASVs of the picophytoplankton (size: 0.2–3 μm; **A**) and micro- and nanophytoplankton (size >3 μm; **B**) communities, respectively. LA, altitude <1,000 m; MA, 1,000 m < altitude <2,000 m; HA, altitude >2,000 m.

The highest gamma diversity was also observed for the lowest altitudinal group (Figures 2E,F). The bipartite association network analysis revealed that of the detected 362 picophytoplankton ASVs, only 84 ASVs were present in all three altitudinal groups. Meanwhile, among the 411 micro- and nanophytoplankton ASVs, there were 200 ASVs in all three altitudinal groups (Figure 3). Compared with lower altitudinal groups, a much lower number of unique picophytoplankton ASVs were found in higher altitudinal groups (Figure 3A). Moreover, much lower shared picophytoplankton ASVs were observed between HA and LA as well as between HA and MA than between MA and LA (Figure 3A). For the micro- and nanophytoplankton, the highest unique ASVs were also found in the lowest altitudinal group (Figure 3B).

### 3.3 The variability of phytoplankton assemblages in the Xijiang headwater streams

**Figure 4:**
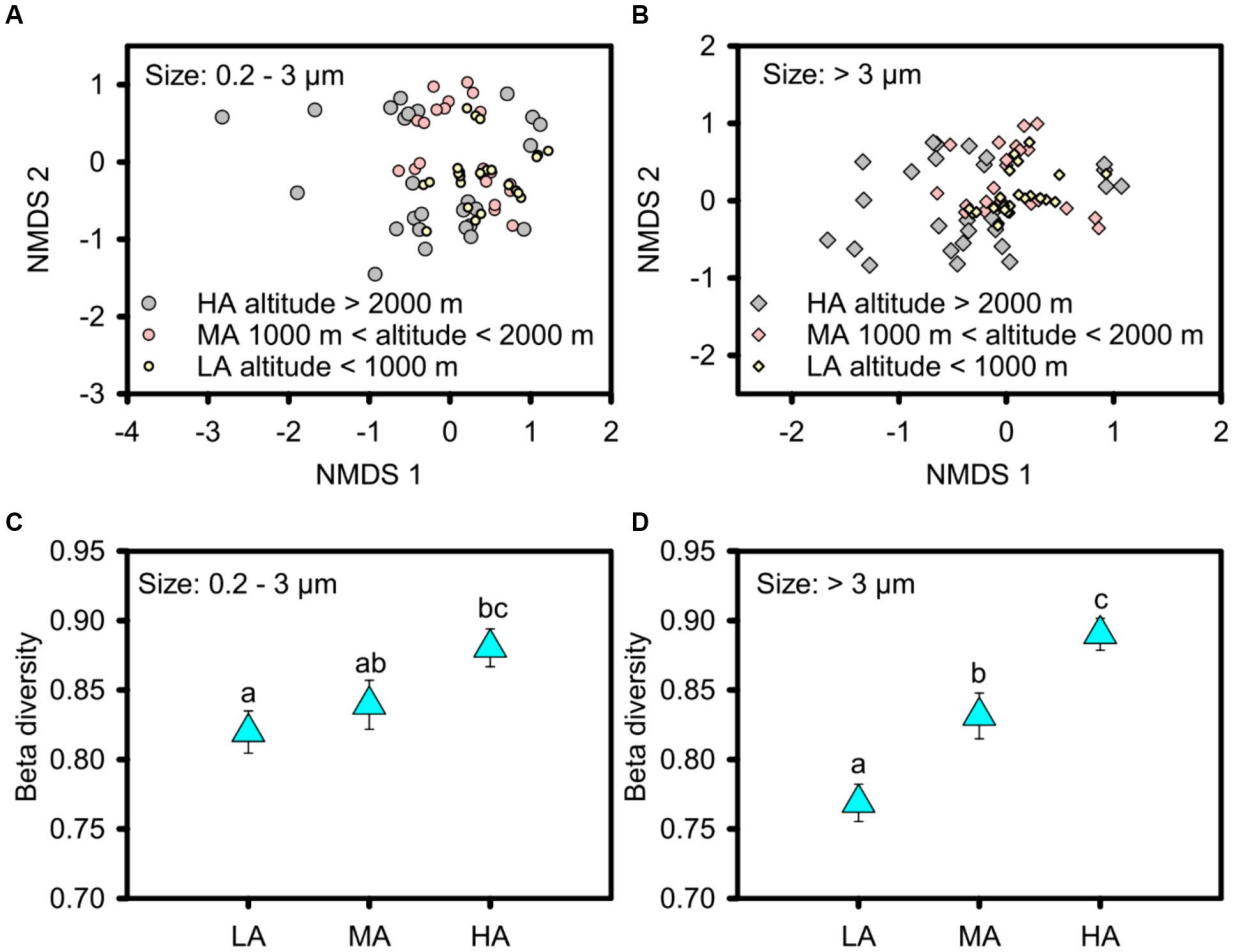
Non-metric multidimensional scaling (NMDS) ordinations showing the beta diversity of the picophytoplankton [size: 0.2–3 μm, **(A,C)**] and micro- and nanophytoplankton [size >3 μm, **(B,D)**] communities in the three altitude groups. Significant differences in beta diversity in the three altitude groups were tested by multivariate homogeneity of group dispersions (variances). LA, altitude <1,000 m; MA, 1000 m < altitude <2000 m; HA, altitude >2000 m. Significant differences (*p* < 0.05) among groups are indicated by different alphabetic letters above the bars.

In contrast to alpha diversity, phytoplankton beta diversity was significantly higher in the higher altitudinal groups for the overall picophytoplankton (permutation test of multivariate homogeneity of group dispersions: *F* = 3.905, *p* < 0.05; Figures 4A,C) as well as for micro- and nanophytoplankton (permutation test of multivariate homogeneity of group dispersions: *F* = 9.358, *p* < 0.01; Figures 4B,D). For the dominant phytoplankton taxa, including *Bacillariophyceae* of Bacillariophyta, *Cryptomonadaceae* of Cryptophyta, *Microcoleus*, *Pseudanabaena*, *Chamaesiphon*, *Phormidium* and *Leptolyngbya* of cyanobacteria and *Euglenaceae* of Euglenophyta, only *Chamaesiphon* in the picophytoplankton exhibited a similar beta diversity pattern to the overall phytoplankton pattern with higher beta diversity in the higher altitudinal groups (Supplementary Figure S5), while the beta diversity of the micro- and nanophytoplankton, including *Bacillariophyceae*, *Microcoleus* and *Chamaesiphon*, all being significantly higher in the higher altitudinal groups (Supplementary Figure S6).

Significant differences in overall phytoplankton structure were observed across the three altitudinal groups for both the overall picophytoplankton (permutation MANOVA: *F* = 2.552, *p* < 0.05; Table 1) and the overall micro- and nanophytoplankton (permutation MANOVA: *F* = 2.976, *p* < 0.01; Table 1). Between the pairwise altitudinal groups, the overall picophytoplankton and overall micro- and nanophytoplankton structure also exhibited significant differences (pairwise permutation MANOVA: *F* = 2.007, *p* < 0.05; Table 1). Of the three altitudinal groups, there were also significant structure differences in specific picophytoplankton clades, such as *Microcoleus*, *Chamaesiphon*, *Phormidium* and *Leptolyngbya* (permutation MANOVA: *F* > 1.633, *p* < 0.05; Supplementary Table S1), and in specific micro- and nanophytoplankton clades, including *Bacillariophyceae*, *Cryptomonadaceae*, *Microcoleus*, *Chamaesiphon*, *Phormidium* and *Leptolyngbya* (permutation MANOVA: *F* > 1.661, *p* < 0.05; Supplementary Table S1).

**Table 1:** Pairwise permanova of picophytoplankton (size: 0.2–3 μm) and micro- and nanophytoplankton (size >3 μm) community structure based on Bray–Curtis dissimilarity.

Groups	Picophytoplankton		Micro- and nanophytoplankton	
	F	*p*	F	*p*
Whole	2.552	0.001**	2.976	0.001**
LA vs. MA	2.007	0.028*	3.153	0.003**
LA vs. HA	2.862	0.001**	3.387	0.003**
MA vs. HA	2.718	0.001**	2.427	0.006**

*p < 0.05; **p < 0.01.

LA, altitudes <1,000 m; MA, 1,000 m < altitudes <2,000 m; HA, altitudes >2,000 m.

### 3.4 The assembly processes of phytoplankton in the streams

**Figure 5:**
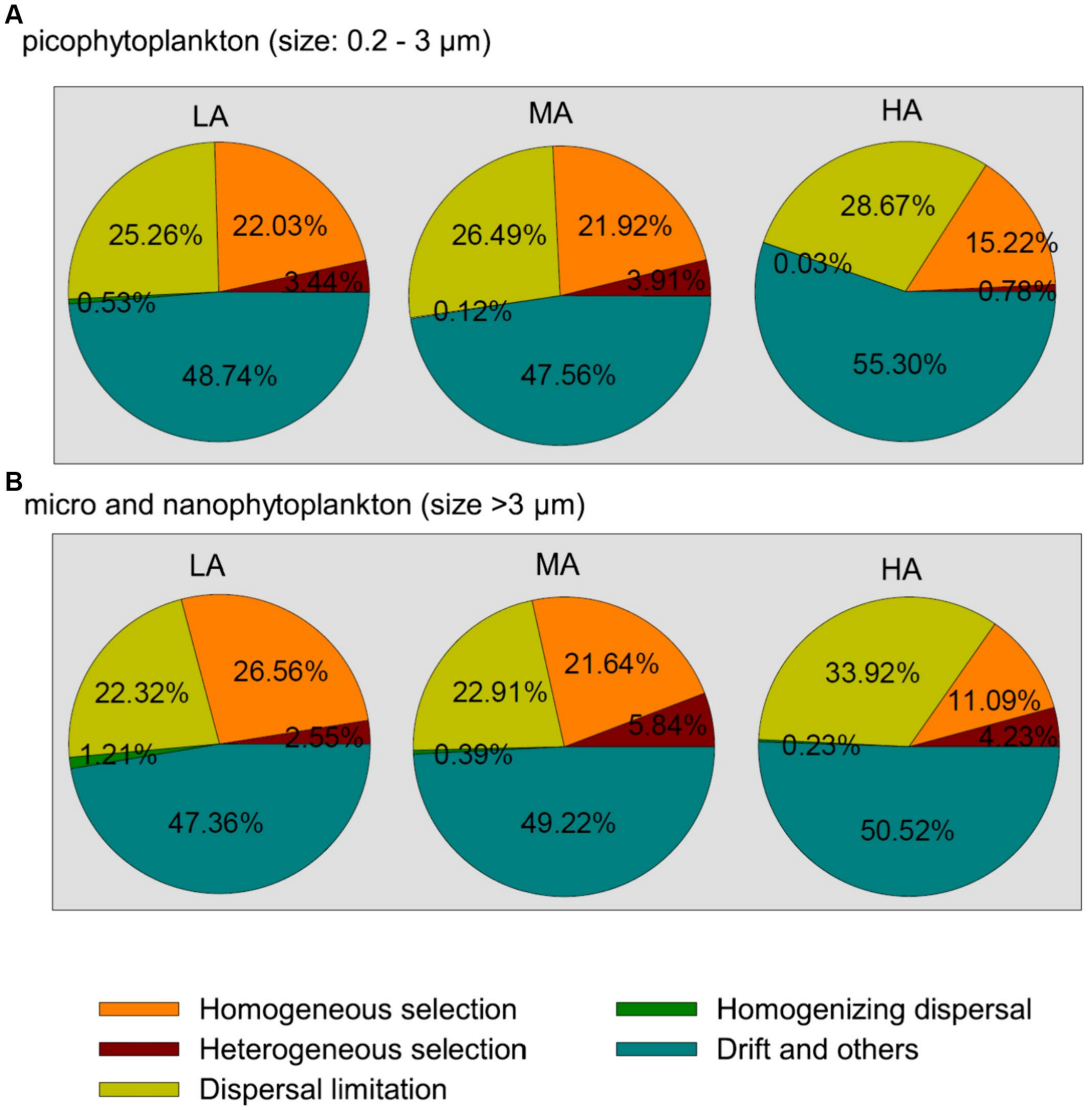
The community assembly processes of the picophytoplankton [size: 0.2–3 μm, **(A)**] and micro- and nanophytoplankton [size >3 μm, **(B)**] communities in the three altitude groups. LA, altitude <1,000 m; MA, 1,000 m < altitude <2,000 m; HA, altitude >2,000 m.

Among the deterministic and stochastic assembly processes (homogeneous and heterogeneous selection, dispersal limitations, homogenizing dispersal and the drift and other fractions), the drift and other fractions were found to contribute the largest fraction to the community assembly of both picophytoplankton and micro- and nanophytoplankton, followed by dispersal limitations and homogeneous selection (Figure 5). For both the picophytoplankton and the micro- and nanophytoplankton, a lower relative importance of homogeneous selection was detected for higher altitudinal groups (Figure 5), indicating that the relative importance of deterministic processes in shaping the phytoplankton assemblages significantly decreased in higher altitudinal groups. However, the processes of dispersal limitations increased in relative importance in shaping the micro- and nanophytoplankton assemblages in higher altitudinal groups (Figure 5).

When relating environmental variables to phytoplankton structure, we found, among the investigated 19 environmental factors, that the picophytoplankton composition was significantly related to stream altitude, ORP and SiO_3_^2−^ concentrations, while the composition of the micro- and nanophytoplankton was significantly related to stream altitude, stream depth and ORP (Supplementary Figure S7). However, in the different altitudinal groups, both the picophytoplankton and the micro- and nanoplankton community structure was significantly related to different combinations of environmental variables (Supplementary Figure S8). Furthermore, multiple regressions on distance matrices (MRM) using permutation tests (Table 2) revealed that the pure effect of environmental variables in explaining both the picophytoplankton and the micro- and nanophytoplankton structure remarkably decreased in the higher altitude groups, while the pure effects of spatial factors increased in the higher altitudinal groups (Table 2). The largest fraction of both the picophytoplankton and the micro- and nanophytoplankton structure could not be explained by the investigated environmental variables or the spatial factors (Table 2).

**Table 2:** Multiple regression on distance matrices (MRM) using permutation tests of picophytoplankton (size: 0.2–3 μm) and micro- and nanophytoplankton (size >3 μm) community structure by environmental (E.) and spatial (S.) factors and their mixed effect (Mix.).

Index	Picophytoplankton			Micro- and nanophytoplankton		
	LA	MA	HA	LA	MA	HA
Pure E.	2.28	8.12	0.14	33.12	16.39	0.87
Pure S.	0.96	8.16	21.68	2.5	8.47	17.41
Mix.	7.95	5.11	4.31	1.7	7.43	6.42
Residuals	88.81	78.61	73.87	62.68	67.71	75.3

### 3.5 The vulnerability of phytoplankton assemblages in the streams

Co-occurrence networks were performed to examine the coexistence of diverse phytoplankton in the streams in the different altitudinal groups (Table 3; Supplementary Figure S9). In a co-occurrence network, the phytoplankton ASVs are referred to nodes, while the connections between the nodes are called edges. In the picophytoplankton network, there were 92 nodes and 479 links for the LA, 64 nodes and 252 links for the MA and 50 nodes and 300 links for the HA (Table 3; Supplementary Figure S9). However, in each altitudinal group, more nodes and links were observed in the micro- and nanophytoplankton than for picophytoplankton. Thus, we observed a network of 196 nodes and 803 links for LA, a network of 113 nodes and 344 links for MA and a network of 112 nodes and 663 links for HA in the micro- and nanophytoplankton (Table 3; Supplementary Figure S9). As for the three altitudinal groups, we found that the co-occurrence networks in the higher altitudinal groups tended to have fewer nodes, fewer links and fewer positive correlations. Both picophytoplankton and micro- and nanophytoplankton co-occurrence networks had a lower diameter, a lower network radius, a lower characteristic path length, a lower network heterogeneity, lower connected components and lower modularity (i.e., the degree that a network can be divided into communities or modules) in the higher altitudinal groups than in the other two groups (Table 3).

**Table 3:** Major topological properties of the observed picophytoplankton (pico) and micro- and nanophytoplankton (micro- and nano) co-occurrence networks in the headwater streams of Xijiang River for three altitudinal groups and their associated random networks.

Sample	Pico observed networks			Pico random networks			Micro- and nano observed networks			Micro- and nano random networks		
	LA	MA	HA	LA	MA	HA	LA	MA	HA	LA	MA	HA
No. of ASVs	413	413	413	413	413	413	413	413	413	413	413	413
No. of nodes	92	64	50	92	64	50	196	113	112	196	113	112
No. of edges	479	252	300	479	252	300	803	344	663	803	344	663
Average number of neighbors	17.63	8.76	18.78	10.41	7.875	12.00	8.79	8.23	10.37	8.19	6.08	11.84
Network diameter	5	4	2	3	4	3	11	11	7	5	6	4
Network radius	3	2	1	3	3	2	6	6	4	3	4	3
Average path length	1.725	1.969	1.304	2.148	2.207	1.792	4.41	4.137	2.582	2.72	2.788	2.152
Clustering coefficient	0.719	0.606	0.843	0.104	0.132	0.239	0.528	0.548	0.728	0.041	0.033	0.109
Network density	0.477	0.351	0.696	0.114	0.125	0.245	0.078	0.161	0.221	0.042	0.054	0.107
Network heterogeneity	0.557	0.57	0.304	0.277	0.295	0.236	0.667	0.723	0.712	0.339	0.386	0.278
Network centralization	0.324	0.357	0.328	0.085	0.084	0.128	0.146	0.199	0.28	0.035	0.054	0.084
Connected components	9	7	6	1	1	1	8	10	6	1	1	1
Modularity	0.493	0.675	0.225				0.762	0.746	0.519			

LA, low altitudes (altitudes <1,000 m); MA, intermediate altitudes (1,000 m < altitudes <2,000 m); HA, high altitudes (altitudes >2,000 m).

**Figure 6:**
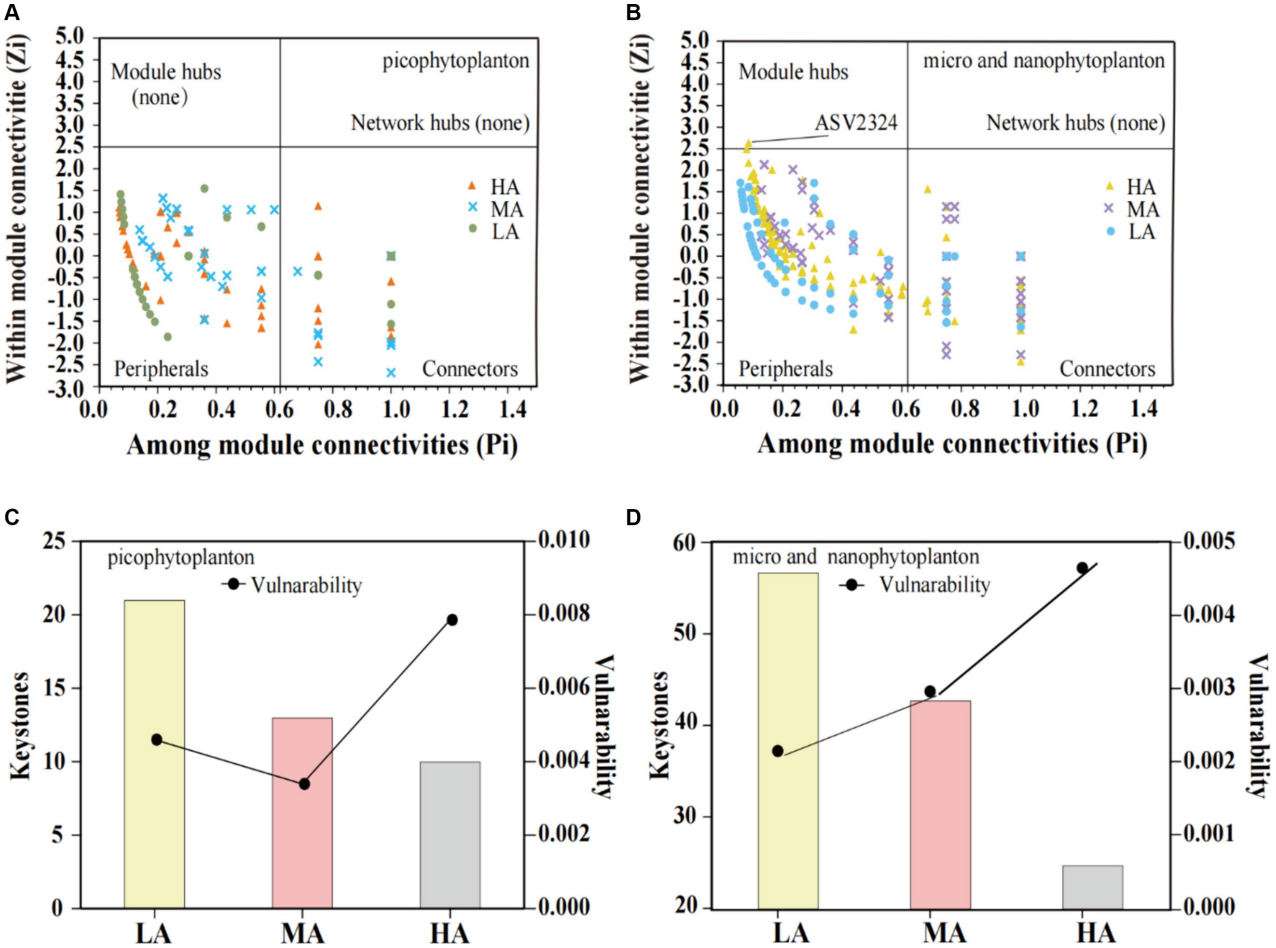
Scatter plot of within-module connectivity (Zi) and among-module connectivity (Pi) showing the distribution of the picophytoplankton [size: 0.2–3 μm, **(A)**] and micro- and nanophytoplankton [size >3 μm, **(B)**] based on their topological roles. The number of keystone taxa and vulnerability of the picophytoplankton [size: 0.2–3 μm, **(C)**] and micro- and nanophytoplankton [size >3 μm, **(D)**] networks in the three altitude groups. LA, altitude <1,000 m; MA, 1,000 m < altitude <2,000 m; HA, altitude >2,000 m.

In both the picophytoplankton and the micro- and nanophytoplankton, the majority of ASVs were nodes connected in modules with few outside, called peripheral (picophytoplankton: 77.2% for LA, 79.7% for MA, and 80.0% for HA; micro- and nanophytoplankton: 71.9% for LA, 62.0% for MA and 77.7% for HA, Figures 6A,B). Among these peripherals, all ASVs had links inside their modules. In the picophytoplankton networks, connectors (i.e., nodes that connect modules) occupied 22.8% ASVs for LA, 20.3% ASVs for MA and 20.0% ASVs for HA, respectively. In the micro- and nanophytoplankton networks, connectors occupied 27.4% ASVs for LA, 38.1% ASVs for MA and 22.3% ASVs for HA, respectively. In the micro- and nanophytoplankton network, the ASV2324 (belonging to cyanobacteria, Figure 6B) was identified as module hubs, having particularly strong associations with most of nodes in the module. We further observed that for both picophytoplankton and micro- and nanophytoplankton, the number of keystone taxa decreased with increasing altitude (picophytoplankton: 21 for LA, 13 for MA and 10 for HA, respectively; micro- and nanophytoplankton: 57 for LA, 43 for MA and 25 for HA, respectively). Moreover, for both the picophytoplankton and the micro- and nanophytoplankton networks, the highest network vulnerability was observed in the HA groups, indicating that HA phytoplankton networks were less stable than those of LA and MA (Figures 6C,D).

## 4 Discussion

Phytoplankton is a polyphyletic group, which has been used as a paradigm system for studying species coexistence since the publication of Hutchinson’s “The Paradox of the Plankton” (Hutchinson, 1961). We found high phytoplankton diversity in the headwater streams of Xijiang River basin. Common freshwater phytoplankton, including cyanobacteria, Bacillariophyta, Chlorophyta, Rhodophyta, Chrysophyta, Euglenophyta, Glaucophyta, Phaeophyta and Cryptophyta, were all detected in both picophytoplankton and micro- and nanophytoplankton. The regional coexistences of diverse phytoplankton communities in mountain streams might be explained by climatic variation, niche diversification, and the recruitment of phytoplankton from the local benthic habitats (Naselli-Flores et al., 2003b; Becker et al., 2008; Rahbek et al., 2019b; Trew and Maclean, 2021). In the Xijiang River basin, headwater stream habitats cover a broad altitudinal gradient spanning from approximately 320 to 2,220 m. Within narrow spatial ranges and across regional-scale habitats, there are not only heterogeneous climatic habitats for phytoplankton assemblage, but also heterogeneity in environmental, hydrological, and mineralogical conditions (Supplementary Figure S1). The differentiation of environmental conditions might benefit the aggregations of diverse phytoplankton in both local and regional scales (Naselli-Flores et al., 2003b; Rahbek et al., 2019a,b). These diverse centers of endemism among headwater streams might support the coexistences of diverse phytoplankton across regional scale habitats (Naselli-Flores et al., 2003a; Stomp et al., 2011; Vallina et al., 2014).

Headwater streams as ours may not only have rapid speciation of taxa but also act as environmental filters (areas with especially high rates of emigration or extinction) (Rahbek et al., 2019b; Brighenti et al., 2021; Chang et al., 2021). We found that phytoplankton diversity decreased monotonically with increasing altitude at a rate of 9 ASV per km for picophytoplankton, and 25 ASV per km for micro- and nanophytoplankton. Headwater streams at higher altitudes served as a filter for microbes, as they had lower alpha and gamma diversity and less unique phytoplankton taxa. Monotonically decreasing altitudinal patterns of microorganisms have also been observed in previous studies, examples being the species diversity of cyanobacterial communities in subarctic ponds in Finland and Norway (Teittinen et al., 2017), biofilm bacterial diversity in streams throughout New Zealand (Lear et al., 2013) and sediment bacterial diversity in subtropical lakes in China (Zeng et al., 2016).

We found key variables that likely co-varied with altitude in explaining the altitude effects on phytoplankton (Supplementary Figure S1). For instance, temperature, which has direct linkages with altitude, is the key factor in the metabolic theory of ecology (Brown et al., 2004; Allen and Gillooly, 2007; Gillooly and Allen, 2007). Thus, metabolic processes influence microbial growth and intraspecific and interspecific competition, and they probably enhance the rate of speciation (Brown et al., 2004). Positive correlation between temperature and diversity has been found for both freshwater and marine phytoplankton in large-scale research investigations (Segura et al., 2015; Righetti et al., 2019). Reduced dispersal might be another mechanism by which phytoplankton diversity may decrease at higher altitudes (Verreydt et al., 2012; Xing and Ree, 2017; Rahbek et al., 2019b). Mountains between headwater streams constitute natural geographical barriers that reduce dispersal for microbes (Chang et al., 2021; Schmeller et al., 2022). Thus, microbial communities in mountain streams have low immigration rates, especially in areas with low precipitation (Schmeller et al., 2022). In our study area, precipitation decreases with increasing altitude (Zeng and Han, 2020b), potentially resulting in weaker microbial dispersal among headwater streams and lower species diversity at higher altitudes. Meanwhile, microorganisms with small cell size are expected to disperse more widely than the larger ones (Gaston et al., 2000). Therefore, compared with picophytoplankton, the larger-sized micro- and nanophytoplankton may have a higher dispersal limitation, explaining the higher slope of linear decreasing relationships between micro- and nanophytoplankton alpha diversity and altitudes than we found for picophytoplankton. In addition, the species diversity of phytoplankton might also be reduced by a shorter growing season at higher altitudes by excluding species appearing during the seasonal succession (Klausmeier, 2010). Moreover, at higher altitudes, the seasonal changes in environmental variables increase, potentially leading to loss of phytoplankton species by filtering of sensitive taxa with a narrow range of environmental tolerance (Currie et al., 2004). Finally, the high UV radiation at higher altitudes may threaten UV-sensitive species of phytoplankton, thus reducing its diversity (Callieri et al., 2001).

The altitudinal diversity pattern of phytoplankton was found to be related to the changes in the balance between homogeneous and heterogeneous selection, dispersal limitations, homogenizing dispersal and stochastic drift (Rahbek et al., 2019b; Wang et al., 2021, 2022; Pritsch et al., 2023). Drift was found to dominate community assembly of both picophytoplankton and micro- and nanophytoplankton in our headwater streams, suggesting that stochastic drift contributes to the non-equilibrium nature of the phytoplankton, thus supporting a high phytoplankton diversity in headwater streams. Moreover, we found that the relative importance of homogeneous selection decreased in shaping both the picophytoplankton and the micro- and nanophytoplankton assemblages at higher altitudes, whereas dispersal limitations increased in the importance for micro- and nanophytoplankton assemblages at higher altitudes. The decreased homogeneous selection at higher altitudes might be due to the complex terrain, high climatic unpredictability as well as increased heterogeneity of environmental variables as shown in the Supplementary Figure S1 and in previous studies (Maloufi et al., 2016; Teittinen and Virta, 2021). The increased dispersal limitations for micro- and nanophytoplankton assemblages at higher altitudes may be ascribed to the enhanced “isolated island” effect of mountain headwater streams and the lower precipitation at higher altitudes at the Xijiang River (Zeng and Han, 2020a,b). The biodiversity of headwater stream phytoplankton was thus characterized by higher variability (both picophytoplankton and micro- and nanophytoplankton) at higher altitudes, where lower homogeneous selection was found than at lower altitudes (Stomp et al., 2011). In addition, more phytoplankton clades showed higher variability at higher altitudes for micro- and nanophytoplankton (i.e., *Bacillariophyceae* and *Microcoleus*) than for picophytoplankton (i.e., only *Chamaesiphon*). This might be explained by higher dispersal limitation and lower homogeneous selection of micro- and nanophytoplankton than of picophytoplankton at higher altitudes (Figure 5).

Besides higher variability, we also found a higher vulnerability of the phytoplankton network structure at higher altitudes, indicating that the decreased homogeneous selection at higher altitudes also resulted in decreased stability of coexisting phytoplankton taxa. Moreover, the higher vulnerability of the coexisting phytoplankton taxa was found to be accompanied by various indices of lower network complexity, such as fewer node and link numbers, lower network heterogeneity, fewer connected components, fewer keystone species and lower modularity. Previous studies have revealed ecological importance of node and link numbers, keystone species, modularity and connectance in maintaining network stability and vulnerability in response to disruptions (Dunne et al., 2002; Grilli et al., 2016; Yuan et al., 2021). These results may reflect that complexity of communities begets its stability (MacArthur, 1955).

## 5 Conclusion

We found high phytoplankton diversity in the studied headwater streams. However, the general high abundance, small size, fast population growth and long-range dispersal of phytoplankton was not sufficient to avoid altitudinal constrains in phytoplankton assemblages. Phytoplankton alpha diversity exhibited a monotonic decreasing relationship with altitude. Such a diversity pattern across the altitudinal gradient is shaped by the balance of selection, dispersal and stochastic drift fractions. High altitudes amplified the “isolated island” effect of the headwater streams, and both the picophytoplankton and the micro- and nanophytoplankton assemblages at higher altitudes was composed of taxa influenced by lower homogeneous selection and higher dispersal limitation. Therefore, the phytoplankton assemblages at higher altitudes exhibited higher variability and vulnerability. These findings are essential for increasing our understanding of aquatic biodiversity in headwater streams, and we encourage long-term and large-scale biodiversity surveys to be conducted to better explore the impacts of environmental changes, such as climate warming, on aquatic biodiversity.

## Data availability statement

The obtained sequencing data in this study were deposited into the Sequence Read Archive (SRA) of the National Center for Biotechnology Information (https://www.ncbi.nlm.nih.gov/) database under accession number PRJNA1003967 (TaxID: 1169740). Other data that support the findings of this study will be made available from the corresponding author on reasonable request.

## Author contributions

YP: Data curation, Formal analysis, Investigation, Methodology, Writing – original draft. CW: Data curation, Formal analysis, Investigation, Methodology, Writing – original draft. GM: Data curation, Formal analysis, Investigation, Writing – original draft. HC: Formal analysis, Writing – review & editing. QW: Funding acquisition, Writing – review & editing. DH: Formal analysis, Writing – review & editing. EJ: Conceptualization, Funding acquisition, Writing – review & editing. LR: Conceptualization, Funding acquisition, Project administration, Supervision, Visualization, Writing – original draft, Writing – review & editing.

## Footnotes

^1^ https://www.R-project.org
