# Supplementary material for: Insight into diversity change, variability and co-occurrence patterns of phytoplankton assemblage in headwater streams: a study of the Xijiang River basin, South China
Source: Front Microbiol. 2024 Aug 19;15:1417651. doi: 10.3389/fmicb.2024.1417651 (PMC11367421; doi:10.3389/fmicb.2024.1417651)
Supplement: Supplementary file 1 [file Image_1.pdf]

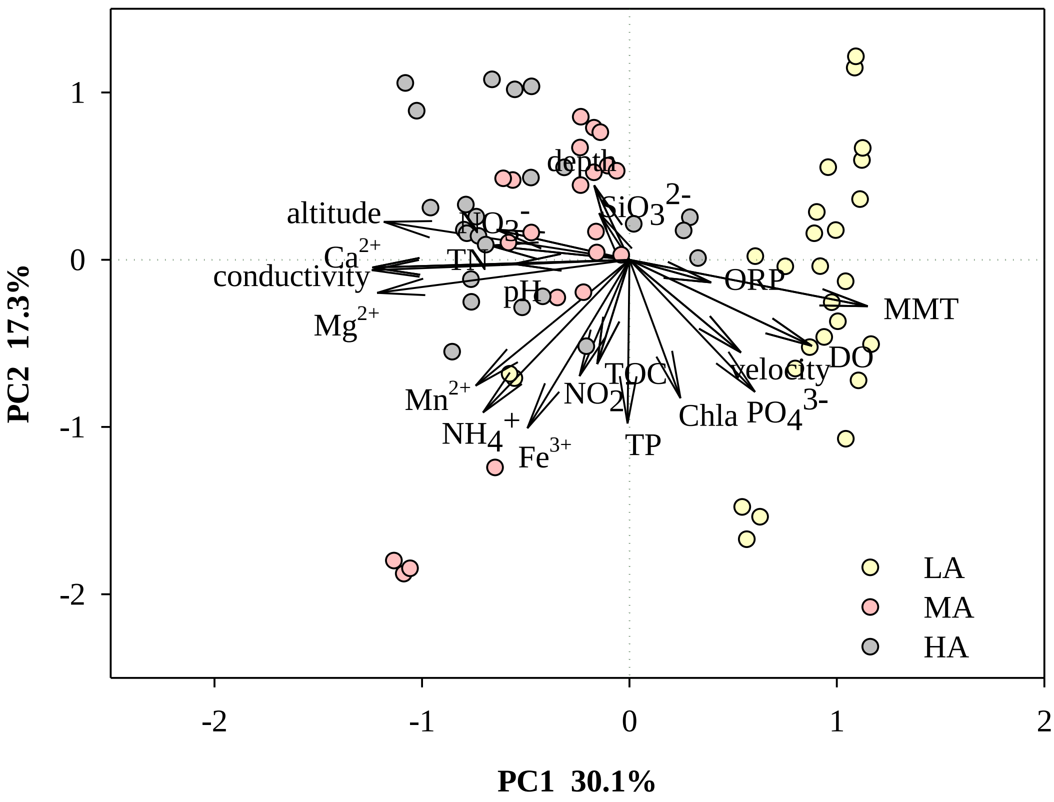

Fig. S1 Biplots of the principal components analysis (PCA) of the environmental factors. MMT, monthly mean temperature; Depth, depth of the stream; velocity, flow velocity; DO, dissolved oxygen; TN, total nitrogen; TOC, total organic carbon; TP, total phosphorus; ORP, oxidation-reduction potential; NO_3^-, nitrate, NO_2^-, nitrite; NH_4^+, ammonia; PO_4^{3-}, orthophosphate; SiO_3^{2-}, silicate; Fe^{3+}, total content of Fe^{3+}; Mn^{2+}, total content of Mn; Ca^{2+}, total content of Ca^{2+}; Mg^{2+}, total content of Mg^{2+}; Chla, chlorophyll *a*. LA: altitude < 1000 m, MA: 1000 m < altitude <2000 m, HA: altitude > 2000 m.
