# Supplementary material for: Insight into diversity change, variability and co-occurrence patterns of phytoplankton assemblage in headwater streams: a study of the Xijiang River basin, South China
Source: Front Microbiol. 2024 Aug 19;15:1417651. doi: 10.3389/fmicb.2024.1417651 (PMC11367421; doi:10.3389/fmicb.2024.1417651)
Supplement: Supplementary file 3 [file Image_3.pdf]

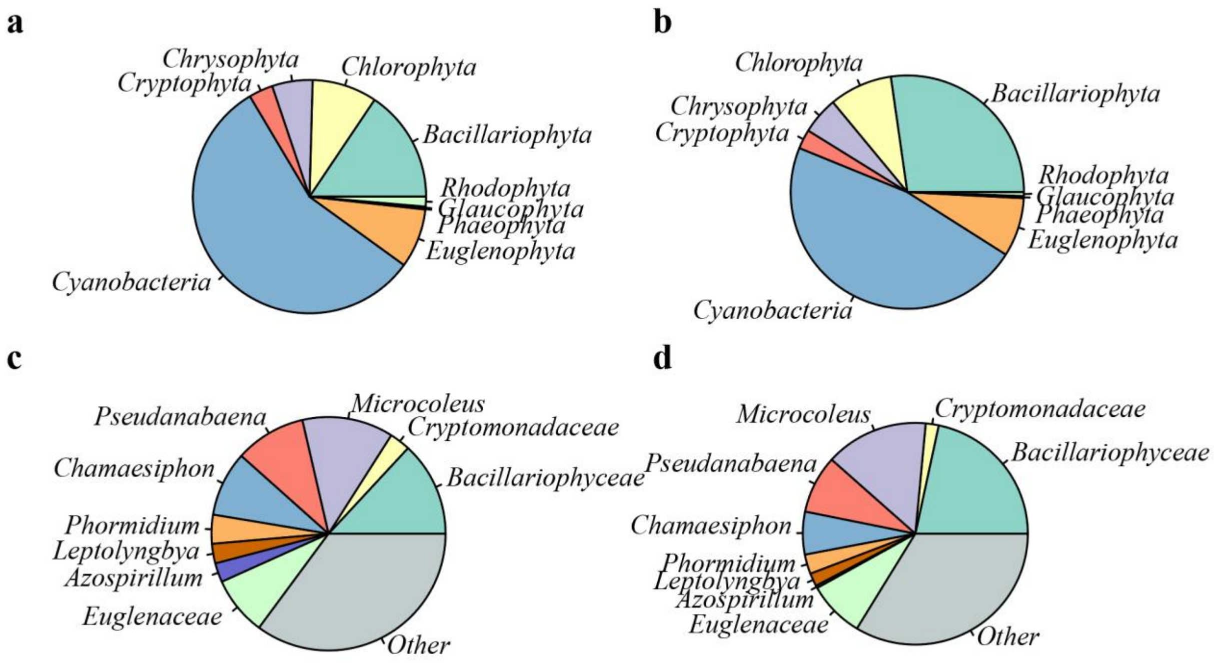

Fig. S3 The composition of dominant phytoplankton phyla (a and b) and clades (c and d) for cell sizes between 0.2 μm and 3 μm (picophytoplankton, a and c) and size > 3 μm (micro- and nanophytoplankton, b and d) in the headwater streams.
