# Supplementary material for: Insight into diversity change, variability and co-occurrence patterns of phytoplankton assemblage in headwater streams: a study of the Xijiang River basin, South China
Source: Front Microbiol. 2024 Aug 19;15:1417651. doi: 10.3389/fmicb.2024.1417651 (PMC11367421; doi:10.3389/fmicb.2024.1417651)
Supplement: Supplementary file 4 [file Image_4.pdf]

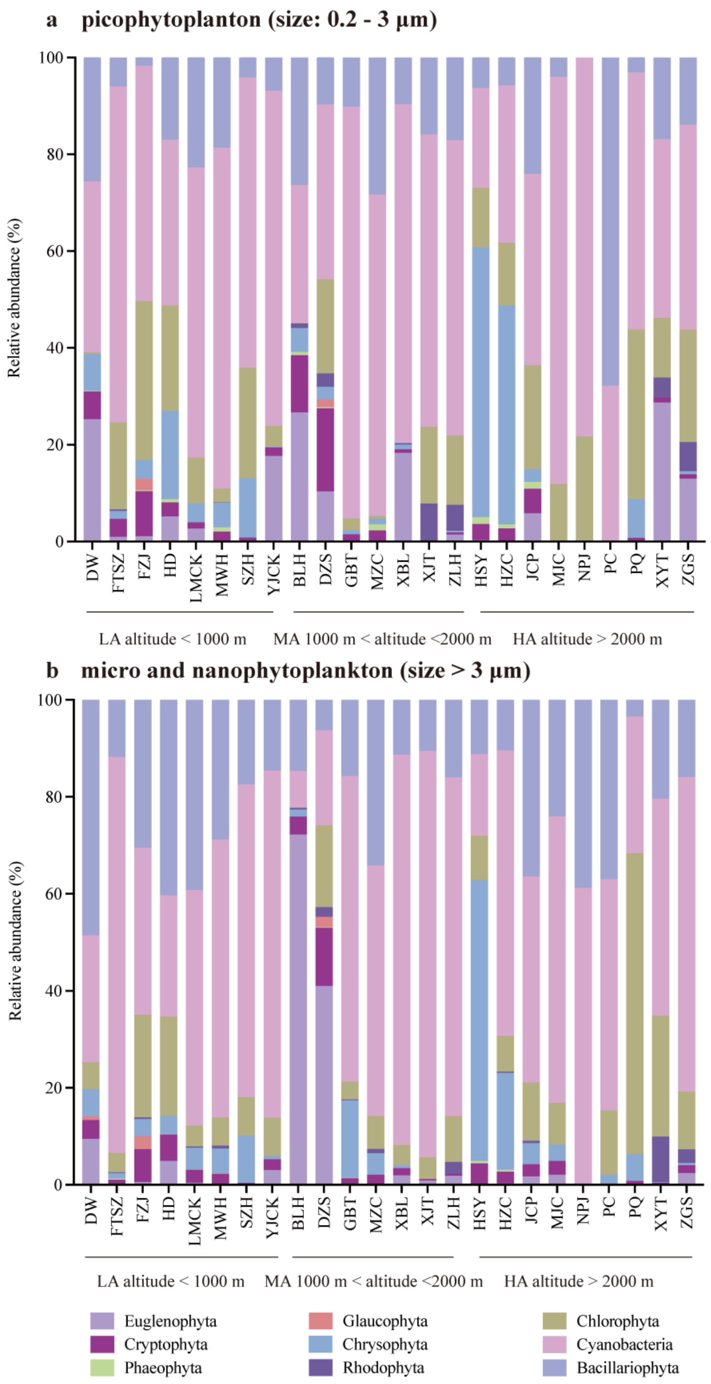

Fig. S4 The distributions of the dominant picophytoplankton (size: 0.2 - 3 µm, a) and micro- and nanophytoplankton (size > 3 µm, b) phyla in the headwaters of the streams. LA: altitude < 1000 m, MA: 1000 m < altitude <2000 m, HA: altitude > 2000.
