# Supplementary material for: Insight into diversity change, variability and co-occurrence patterns of phytoplankton assemblage in headwater streams: a study of the Xijiang River basin, South China
Source: Front Microbiol. 2024 Aug 19;15:1417651. doi: 10.3389/fmicb.2024.1417651 (PMC11367421; doi:10.3389/fmicb.2024.1417651)
Supplement: Supplementary file 6 [file Image_6.pdf]

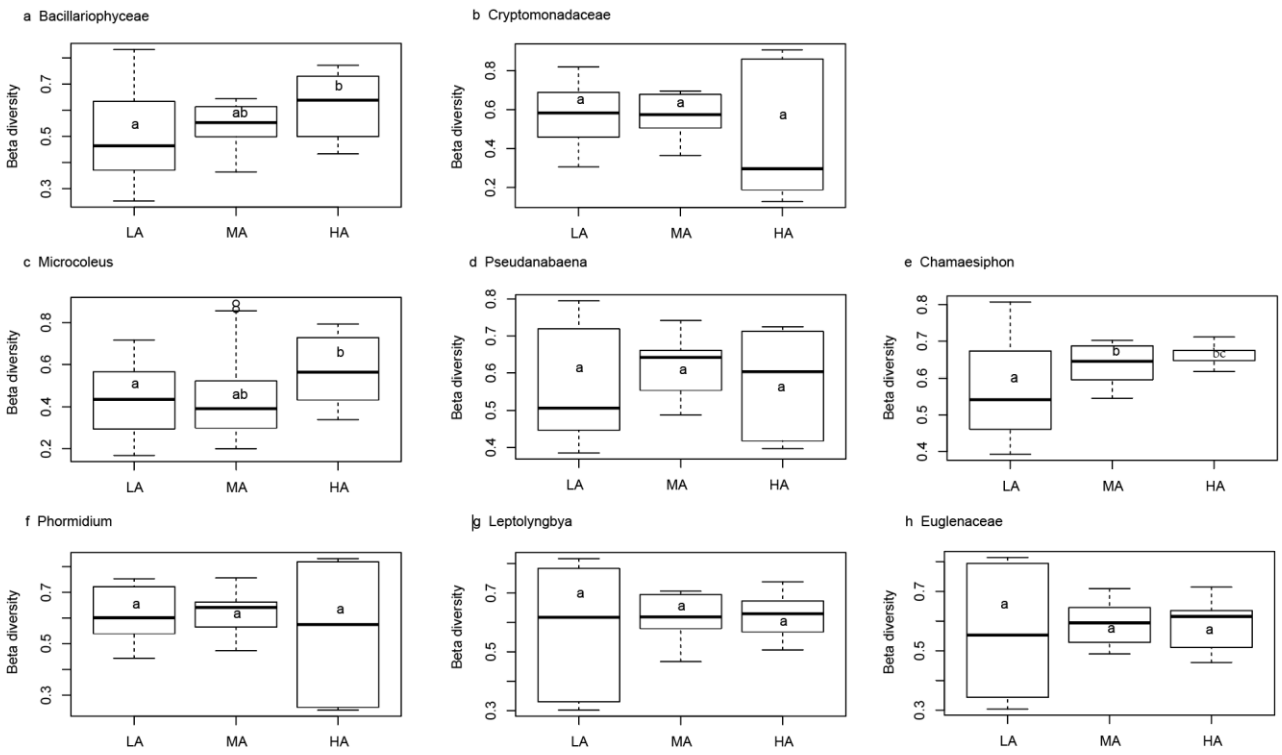

Fig. S6 Beta diversity in micro- and nanophytoplankton communities tested by permutation test and Tukey multiple comparisons for homogeneity of multivariate dispersions. LA: altitude < 1000 m, MA: 1000 m < altitude <2000 m, HA: altitude > 2000 m. Significant ($p < 0.05$) differences among groups are indicated by different alphabetic letters above the bars.
