# Supplementary material for: Insight into diversity change, variability and co-occurrence patterns of phytoplankton assemblage in headwater streams: a study of the Xijiang River basin, South China
Source: Front Microbiol. 2024 Aug 19;15:1417651. doi: 10.3389/fmicb.2024.1417651 (PMC11367421; doi:10.3389/fmicb.2024.1417651)
Supplement: Supplementary file 7 [file Image_7.pdf]

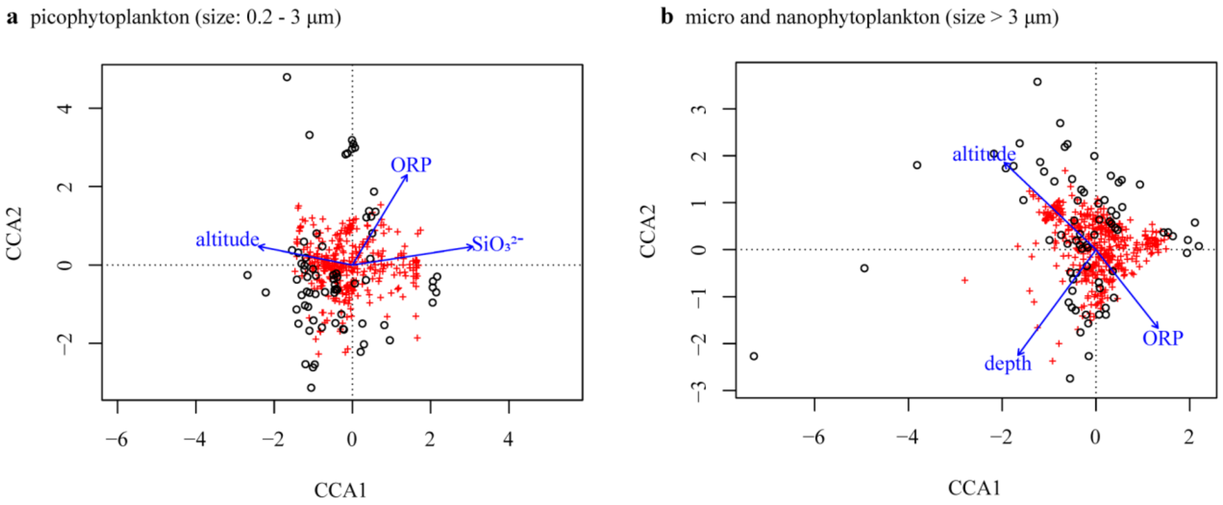

Fig. S7 Canonical correspondence analysis relating the composition of picophytoplankton (size: 0.2 - 3 μm, a) and micro- and nanophytoplankton (size > 3 μm, b) with the investigated environmental factors. Depth, depth of the stream; ORP, oxidation-reduction potential; SiO_3^{2-}, silicate.
