# Supplementary material for: Insight into diversity change, variability and co-occurrence patterns of phytoplankton assemblage in headwater streams: a study of the Xijiang River basin, South China
Source: Front Microbiol. 2024 Aug 19;15:1417651. doi: 10.3389/fmicb.2024.1417651 (PMC11367421; doi:10.3389/fmicb.2024.1417651)
Supplement: Supplementary file 8 [file Image_8.pdf]

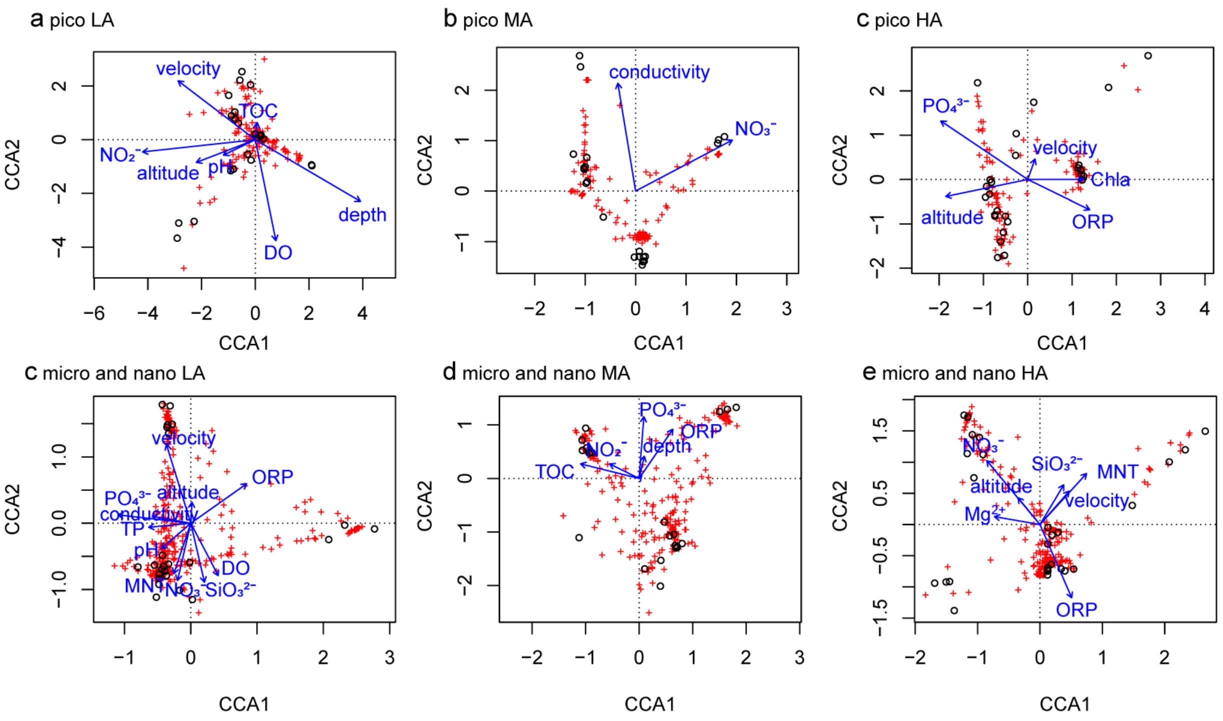

Fig. S8 Correspondence analysis of picophytoplankton (a, b, and c) and micro- and nanophytoplankton (d, e, and f) communities in the three altitudinal groups of headwater streams. LA: altitude < 1000 m, MA: 1000 m < altitude < 2000 m, HA: altitude > 2000 m.
