# Supplementary material for: Insight into diversity change, variability and co-occurrence patterns of phytoplankton assemblage in headwater streams: a study of the Xijiang River basin, South China
Source: Front Microbiol. 2024 Aug 19;15:1417651. doi: 10.3389/fmicb.2024.1417651 (PMC11367421; doi:10.3389/fmicb.2024.1417651)
Supplement: Supplementary file 9 [file Image_9.pdf]

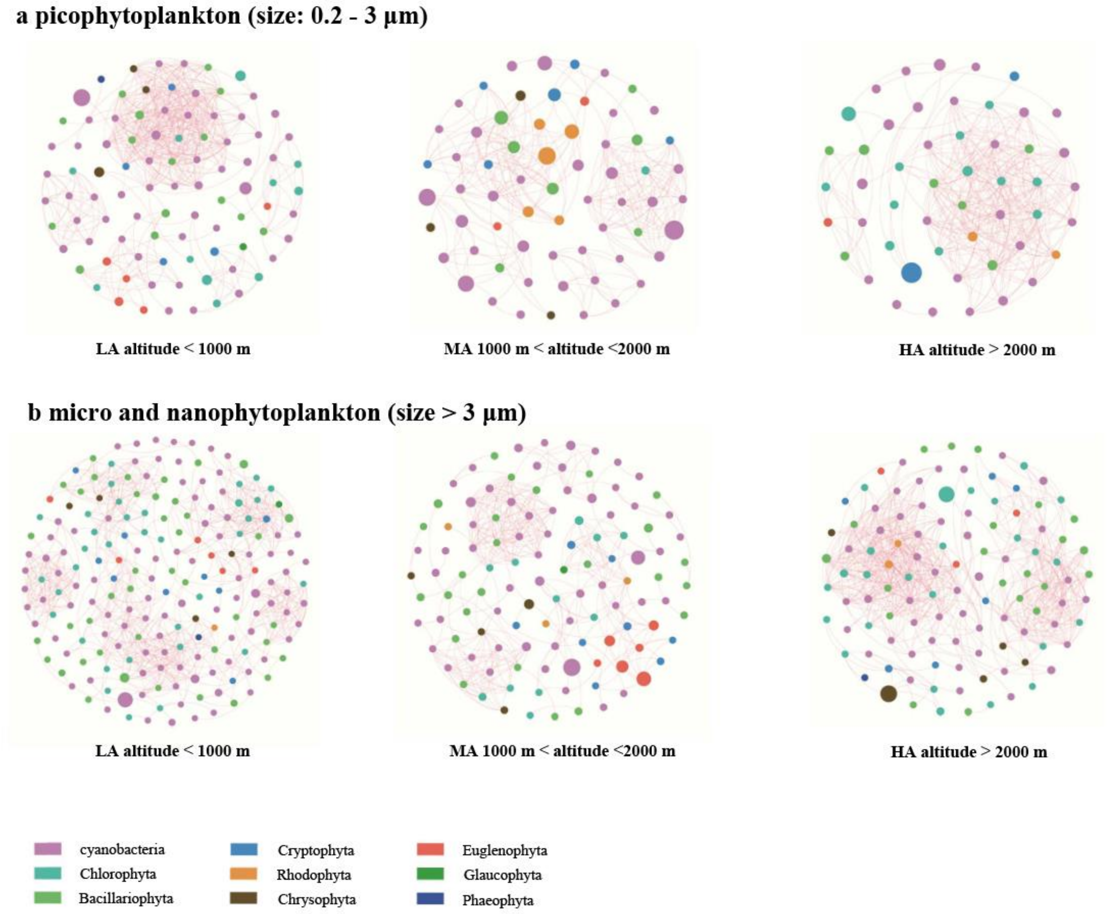

Fig. S9 Co-occurrence network patterns of picophytoplankton and micro- and nanophytoplankton communities for three altitudinal groups of headwater streams. The lines between each pair of nodes represent positive (in pink) interactions with strong and significant correlations. LA: altitudes < 1000 m, MA: 1000 m < altitudes < 2000 m, HA: altitudes > 2000 m.
