# Supplementary material for: Insight into diversity change, variability and co-occurrence patterns of phytoplankton assemblage in headwater streams: a study of the Xijiang River basin, South China
Source: Front Microbiol. 2024 Aug 19;15:1417651. doi: 10.3389/fmicb.2024.1417651 (PMC11367421; doi:10.3389/fmicb.2024.1417651)
Supplement: Supplementary file 10 [file Table_1.docx]

**Table S1** PERMANOVA of phytoplankton community structure based on Bray-Curtis dissimilarity. LA: altitudes < 1000 m, MA: 1000 m < altitudes < 2000 m, HA: altitudes > 2000 m.

| Phylum | Taxa | Picophytoplankton | |  | Micro- and nanophytoplankton | |
| --- | --- | --- | --- | --- | --- | --- |
|  |  | F | *p* |  | F | *p* |
| Bacillariophyta | Bacillariophyceae | 1.323 | 0.186 |  | 2.188 | 0.007** |
| Cryptophyta | Cryptomonadaceae | 1.569 | 0.101 |  | 2.381 | 0.014 * |
| Cyanobacteria | Microcoleus | 2.502 | 0.005 ** |  | 2.986 | 0.002** |
| Cyanobacteria | Pseudanabaena | 1.319 | 0.179 |  | 1.537 | 0.08 |
| Cyanobacteria | Chamaesiphon | 1.633 | 0.039 * |  | 1.661 | 0.019* |
| Cyanobacteria | Phormidium | 2.814 | 0.002 ** |  | 2.056 | 0.004** |
| Cyanobacteria | Leptolyngbya | 2.367 | 0.006 ** |  | 2.277 | 0.002** |
| Euglenophyta | Euglenaceae | 0.698 | 0.677 |  | 1.531 | 0.109 |

**p* < 0.05; ***p* < 0.01.
